# Enhancing Medical Image Denoising with Innovative Teacher–Student Model-Based Approaches for Precision Diagnostics

**DOI:** 10.3390/s23239502

**Published:** 2023-11-29

**Authors:** Shakhnoza Muksimova, Sabina Umirzakova, Sevara Mardieva, Young-Im Cho

**Affiliations:** Department of IT Convergence Engineering, Gachon University, Sujeong-gu, Seongnam-si 461-701, Republic of Korea; shakhnoza02@gachon.ac.kr (S.M.); sevara1998@gachon.ac.kr (S.M.)

**Keywords:** medical image denoising, lightweight model, teacher–student network, model speed optimization

## Abstract

The realm of medical imaging is a critical frontier in precision diagnostics, where the clarity of the image is paramount. Despite advancements in imaging technology, noise remains a pervasive challenge that can obscure crucial details and impede accurate diagnoses. Addressing this, we introduce a novel teacher–student network model that leverages the potency of our bespoke NoiseContextNet Block to discern and mitigate noise with unprecedented precision. This innovation is coupled with an iterative pruning technique aimed at refining the model for heightened computational efficiency without compromising the fidelity of denoising. We substantiate the superiority and effectiveness of our approach through a comprehensive suite of experiments, showcasing significant qualitative enhancements across a multitude of medical imaging modalities. The visual results from a vast array of tests firmly establish our method’s dominance in producing clearer, more reliable images for diagnostic purposes, thereby setting a new benchmark in medical image denoising.

## 1. Introduction

Over the past decades, advancements in technology, especially in computing, the internet, data storage, and wireless systems, have greatly influenced medical imaging and diagnostics. This progress has enriched medical science, enhancing disease diagnosis and treatment. Often, these imaging techniques are used to monitor previously diagnosed and treated conditions. However, medical images can be affected by distortions like noise. This noise might be consistent, like white noise, or vary based on device operation or signal processing [1]. Noise can blur the images, complicating disease detection and potentially leading to severe consequences, including fatalities. Therefore, it is crucial to remove noise from medical images before further analysis [2]. A primary hurdle in medical imaging is capturing images without omitting important details. There is a strong likelihood that images can become distorted by noise or other disruptions during their acquisition or subsequent processing. Noise is essentially the unexpected alteration of the original pixel values. This not only diminishes the image’s clarity but is particularly problematic when imaging small objects with subtle contrasts [3]. Noise in medical images can arise during the imaging process due to environmental factors or issues with the image-capturing system, like sensor sensitivity fluctuations from temperature changes. Removing noise becomes complex when the specific noise type is not identified. Various noise types, including Gaussian, Poisson, salt and pepper (impulse noise), and speckle noise, can disrupt the detailed patterns in images [4].

Traditional methods for denoising after image acquisition fall into three categories: filtering techniques [5], transform domain methods [6], and statistical methods [7]. While basic smoothing filters can effectively remove general noise, they tend to blur images, especially finer details. To retain image sharpness, edge-preserving filters can be employed. Nonlinear filters, like anisotropic diffusion filters [8], are beneficial for maintaining specific anatomical details. The integrity of anatomical borders can be retained using non-local mean filters [9], which utilize both image intensity and structure information from distant regions to estimate and reduce noise. The block-matching 3D filtering (BM3D) technique employs a sparse representation for eliminating noise within a transformed domain [10]. Meanwhile, ref. [11] used a nonparametric empirical Bayesian method to model and remove Rician noise in MR images.

Deep learning, a sophisticated subset of machine learning, has recently made significant strides in the domain of medical image denoising. Leveraging the prowess of convolutional neural networks (CNNs) [12], deep learning models have the aptitude to detect intricate patterns across vast datasets [13]. When exposed to substantial volumes of medical imagery, these models effectively discern between genuine anatomical structures and extraneous noise. Among the variety of deep learning architectures employed for this task, autoencoders stand out [14]. These neural networks aim to replicate their input data, and when trained on noisy medical images, they adeptly learn to output their cleaner versions. The underlying principle is the model’s ability to compress the image into a condensed representation and subsequently reconstruct it, in the process filtering out unwanted noise.

Another significant architectural design used is U-Net [15]. While its initial design catered to biomedical image segmentation, its application has been extended to denoising. The structure of U-Net is particularly beneficial for precise localization, ensuring that while the noise is filtered out, the essential details of the image are retained [16]. One of the primary advantages of employing deep learning for medical image denoising is its adaptability. The models can address various noise types within a single image without the prerequisite of explicit noise categorization. Furthermore, in terms of both subjective visual interpretation and objective metric evaluations, deep learning techniques have shown superiority over traditional denoising methods [17]. Also, their rapid processing speed, once trained, renders them fit for real-time clinical applications [18].

However, challenges persist. Deep learning models are voracious for large, labeled datasets. In the context of medical image denoising, this translates to a necessity for both noisy and the corresponding noise-free images [19]. Another potential pitfall is overfitting, where models become overly attuned to the specific noise characteristics they are trained on, leading to suboptimal performance on unfamiliar noise types [20]. Additionally, the computational demands for training such sophisticated models are substantial. In the rapidly evolving landscape of medical imaging, there is a discernible trend towards hybrid models that synergize traditional algorithms with deep learning, aiming for enhanced denoising outcomes [21]. Concurrently, transfer learning is gaining traction, where models adept in one form of medical imaging, like MRI, are fine-tuned for another, such as CT [22].

The potential of deep learning in the realm of medical image denoising is immense. As algorithms mature and data accessibility grows, deep learning is poised to become an indispensable tool in clinical imaging.

Addressing the challenges of noise in medical images necessitates the creation of efficient denoising models that offer rapid inference speeds. These models should ideally strike a balance between producing high-quality images and ensuring computational efficiency. In the quest for this balance, earlier studies have explored strategies to enhance efficiency. One common approach has been to minimize the number of model parameters, thus streamlining the model’s complexity [23]. Another tactic has been to reduce the number of floating-point operations (FLOPs), which are a direct measure of the computational intensity of an operation. By optimizing these aspects, researchers aim to create models that are both fast and effective, making them more applicable in real-world, time-sensitive clinical settings. Recursive networks that employ weight-sharing strategies have become a popular method to diminish the number of parameters within a model [24]. However, the intricacy of their graph topology means that even with fewer parameters, they might not inherently lead to a reduction in the number of operations or speed up inference time. In parallel, while techniques designed to cut down on FLOPs, such as depth-wise convolutions, feature splitting, and shuffling [25], are commonplace, their impact on computational efficiency is not guaranteed. The actual effectiveness of these techniques can be contingent upon various factors, including the specific architecture in use and the nature of the dataset being processed [26]. Thus, we approach the challenge of crafting an efficient denoising network from a distinct vantage point. Instead of delving into intricate network design modifications, we ponder the feasibility of harnessing more effective training strategies. Our proposition revolves around refining and optimizing the denoising model, primarily through enhanced training methodologies. Central to our approach is the teacher–student model, where we posit that strategic training can lead to efficiency gains without necessitating extensive alterations to the underlying network structure. The motivation behind our research stems from this critical challenge: to enhance the quality of medical images by effectively reducing noise, thereby directly contributing to more accurate and reliable diagnostics. Our proposed solution, a novel teacher–student network model augmented with our advanced NoiseContextNet Block, directly addresses this issue. By focusing on the distinct characteristics of noise in medical images and leveraging advanced machine-learning techniques for noise reduction, we aim to substantially elevate the clarity of medical imaging. This enhancement is not just a technological advancement but a crucial step towards improving patient outcomes, as clearer images facilitate more precise identification of ailments and conditions. Moreover, the iterative pruning technique we introduce is designed to optimize the efficiency of our model, ensuring that our solution is not only effective in enhancing image quality but also viable for real-world application, given the computational constraints in medical imaging environments.

The objective of the proposed research is twofold: to significantly improve the quality of medical images for more accurate diagnostics, and to ensure that these improvements are practical and applicable in clinical settings. Our extensive experiments and results aim to demonstrate not just the theoretical viability of our approach but its practical effectiveness and superiority in addressing one of the most pressing challenges in medical imaging. Additionally, we delve deeper into our methodology by furnishing comprehensive ablation studies. These studies elucidate the impact and efficacy of our chosen architectural designs and experimental decisions. Summing up the pivotal takeaways from our research, the primary contributions of this study are:

- We introduce a novel design based on the teacher–student paradigm, optimizing both the speed and size of the model. This design notably enhances the student network’s learning potential and offers a reshaped concise form upon convergence, utilizing staggered distillation methods for more effective knowledge transfer.
- The introduction of the NoiseContextNet Block, a specialized component tailored for medical imagery. This block adeptly differentiates nuanced anatomical characteristics from noisy areas, ensuring comprehensive context-rich information is transmitted, thereby aiding in precise model outputs.
- We present the attention-based depth-wise convolution network (ADWC) with linear complexity. Pivoting attention from spatial dimensions to channels, this approach produces an insightful attention map by harnessing cross-covariance across channels, making it indispensable for denoising.
- The incorporation of an iterative pruning technique post the reparameterization of the student network. This strategy systematically jettisons the least consequential elements of the network, ensuring that the model’s predictive capabilities are retained while enhancing its efficiency.

## 2. Materials and Methods

**Figure 1:**
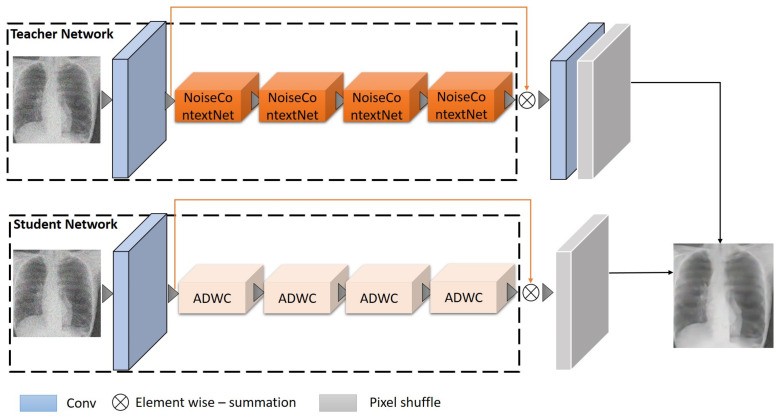
Illustration of the methodology adopted to optimize our model. Starting with a comprehensive teacher network, the smaller student model is guided through knowledge distillation. To enhance the learning capability of the student model, specialized blocks are incorporated to capture intricate details. The student network’s architecture combines sequential and parallel branches, further bolstered by residual connections. This design allows for a more effective and nuanced transfer of knowledge from the teacher to the student model.

We suggest a method to make medical image networks more efficient by reducing noise and enhancing performance through four main steps. In the first step in Section 3.1, we present our design, modeled on a teacher–student approach as depicted in Figure 1. In the next step in Section 3.2, we introduce a technique called NoiseContextNet Block to sharpen the initial image quality. In Section 3.3, we delve into a deep convolution process paired with an attention mechanism, amplifying the network’s efficiency. In the final process in Section 3.4, we implement a repetitive pruning approach to lessen the computational burden of the model.

Algorithm 1 encapsulates the training procedure of the proposed model. It outlines the sequential steps of the process, starting from inputting the noisy images to producing denoised output, and includes the use of specialized blocks and techniques to improve the learning and accuracy of the model.
**Algorithm 1.** Medical Image-Denoising Procedure1: **Input**: Noisy medical images; 2: **Output**: Denoised medical images; 3: **for** number **of** training iterations **do** 4:  Sample batch **of** *n* noisy images {*Inoisy*1, *Inoisy*2, …, *Inoisyn*} **from** dataset *D*; 5:  Pass *Inoisyn* through the initial layer **to** obtain basic feature maps *F*0; 6:  **for each** NoiseContextNet Block **in** the network **do** 7:    Apply the block **to** extract and differentiate contextual features **from** noise; 8:  **end for** 9:  Apply ADWC **to** enhance important features and suppress noise **in** the feature maps; 10:   Concatenate the enhanced features **with** contextual information **from** NoiseContextNet Block; 11:   Apply the iterative pruning technique **to** the concatenated features; 12:   **for each** pruned feature **set do** 13:     Fine-tune the network **to** compensate **for** the pruned elements; 14:   **end for** 15:   Generate denoised image **output** *Idenoisedn*; 16:   Calculate loss between *Idenoisedn* and ground truth clean images; 17:   Update model weights using backpropagation and optimization algorithm; 18: **end for**

### 2.1. Teacher–Student Network

In Figure 1, our methods enhance the model’s speed and size. While it is tricky to train a small model directly, starting with a larger teacher network and then guiding a student network through knowledge distillation is more practical. However, given the vast learning disparity between the teacher and student networks, the student network might struggle to grasp intricate details if directly distilled. To address the noted challenge, we introduced blocks to obtain rich, detailed information, assisting the student network with constrained learning capabilities. The disparity between the teacher and student networks in terms of learning capabilities is significant. The teacher network, with its advanced and complex architecture, is adept at extracting and processing intricate details from medical images. In contrast, the student network, designed to be more streamlined and less resource-intensive, inherently lacks this level of sophistication. This gap poses a challenge: the student network may struggle to replicate the nuanced understanding and detailed feature extraction exhibited by the teacher network. To mitigate this issue, the block-based structure, such as the NoiseContextNet Block, is introduced. This innovative design is key to enriching the learning process of the student network. The blocks are engineered to capture and distill complex patterns and details from the teacher network in a form that is more digestible for the student network. In essence, these blocks act as intermediaries, translating the rich, detailed information from the teacher network into a format that the student network can effectively learn and utilize. Furthermore, the block-based structure serves a dual purpose. It not only aids in the transfer of knowledge but also ensures that the student network, despite its constraints, does not compromise on the quality of output. By incorporating these blocks, the student network is equipped to handle the intricate details necessary for high-quality medical image denoising, bridging the gap between its inherent limitations and the need for precise and accurate image analysis. The block-based structure is a strategic response to the challenges posed by the learning disparity in teacher–student network models. It ensures that the student network can effectively learn from the teacher without being overwhelmed by the complexity of the information, thereby maintaining the integrity and quality of the denoising process.

Using these blocks, we crafted the student network structure with sequential and parallel branches, along with residuals. These added branches amplify the student network’s learning potential. Once the student network reaches convergence, it can be redefined into a more streamlined framework. This action boosts the model’s learning capacity without adding complexity. To leverage the teacher network’s guidance to its fullest, we employ a tiered distillation approach, positioning anchor points at various network nodes and utilizing multi-layered features at these anchors for distillation.

### 2.2. NoiseContextNet Block

**Figure 2:**
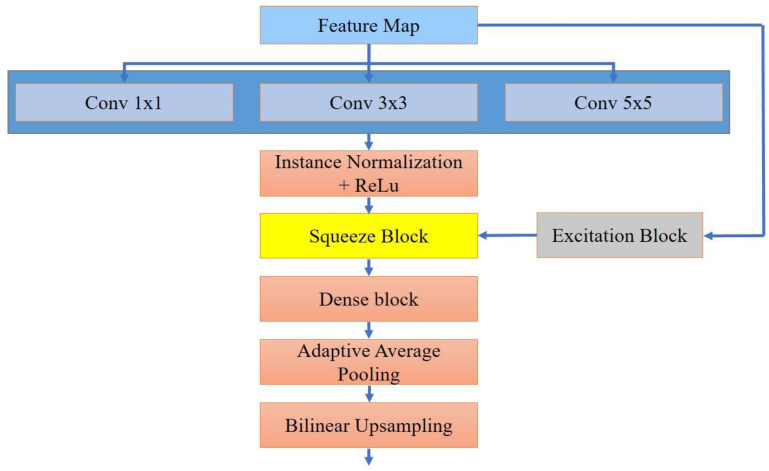
Here, the Noise-ContextNet Block is depicted, a specialized component designed to address noise in medical images. This block enhances the model’s proficiency in distinguishing intricate, important features from areas perturbed by noise, adeptly recognizing contextual relationships within the image.

To better tackle noise in medical images and accentuate the dependencies of the underlying anatomical structures, we present a refined component termed the NoiseContextNet Block Figure 2. This block markedly enhances the model’s ability to determine distinct anatomical features from noise-affected regions by efficiently discerning contextual relationships within those features. 

The NoiseContextNet is intricately designed, incorporating multi-scale convolution layers, a squeeze-and-excitation block, residual connections, a dense block, adaptive average pooling, and bilinear upsampling. We concurrently employ multi-scale convolution layers of dimensions 1 × 1, 3 × 3, and 5 × 5. This arrangement allows the model to perceive features across varied spatial dimensions, extracting both specific and overarching patterns that elevate the quality of the resulting medical image representations. Following these convolution layers, we use instance normalization. By determining mean and variance statistics for each feature map, this normalization allows each medical image to be treated individually, heightening the model’s resilience to contrast disparities and boosting its efficacy in environments where contrast consistency is vital. The strategically positioned squeeze-and-excitation block dynamically recalibrates feature maps. By recognizing correlations between channels, it adaptively adjusts their relevance, improving the model’s diagnostic power. Our NoiseContextNet also features skip connections, which ensure a steady gradient flow across the network, addressing potential gradient issues and optimizing the network’s learning and performance. Instead of typical fully connected layers, a dense block is employed. The unique interconnected structure of this block intensifies the information and gradient flow, leading to enhanced medical image analysis. The NoiseContextNet also integrates 1 × 1 adaptive average pooling, which efficiently narrows down feature map dimensions while retaining vital information. Bilinear upsampling, on the other hand, augments feature map resolution when necessary, preserving detailed filtering out pertinent feature information crucial for precise medical imaging.

The primary objective of the NoiseContextNet is to convey extensive context-rich information across each feature map, equipping the network to discern higher-order contextual attributes. Given the synergistic modifications brought about by adaptive average pooling and residual connections, the NoiseContextNet remains computationally streamlined, yet significantly boosts network learning. The dense block’s pivotal role is in the self-gating system’s excitation process, drawing out that conducts regulated feature modification, specifically filtering out less pertinent features and only permitting valuable data to progress deeper into the network structure. Within our proposed framework, the NoiseContextNet mandate is to transmit comprehensive contextual insights about the valuable feature regions, thus aiding in accurate output.

### 2.3. Attention-Based Depth-Wise Convolution Network

In standard attention network approaches, the computational load from key–query interactions increases at a rate proportional to the square of the image’s spatial resolution. This makes it challenging to utilize the attention network for many medical image-denoising tasks, especially when dealing with high-resolution images. The traditional attention mechanisms often require substantial computational resources due to their focus on spatial dimensions within images. These mechanisms, when applied to the high-dimensional data typical in medical imaging, can become computationally intensive and less feasible for practical applications. The innovation of the ADWC approach lies in its shift of focus from spatial dimensions to channel interactions within the image data. This shift is significant as it allows for a more streamlined processing approach. By concentrating on the relationships between different channels of the image data, ADWC sidesteps the complexity inherent in handling spatial details, which can be particularly resource-intensive in high-resolution medical images. Moreover, the ADWC is designed with linear complexity, a crucial feature that ensures computational resources scale linearly rather than exponentially with the size of the input. This aspect is particularly important in medical imaging contexts where images are not only high-resolution but also need to be processed accurately and swiftly.

**Figure 3:**
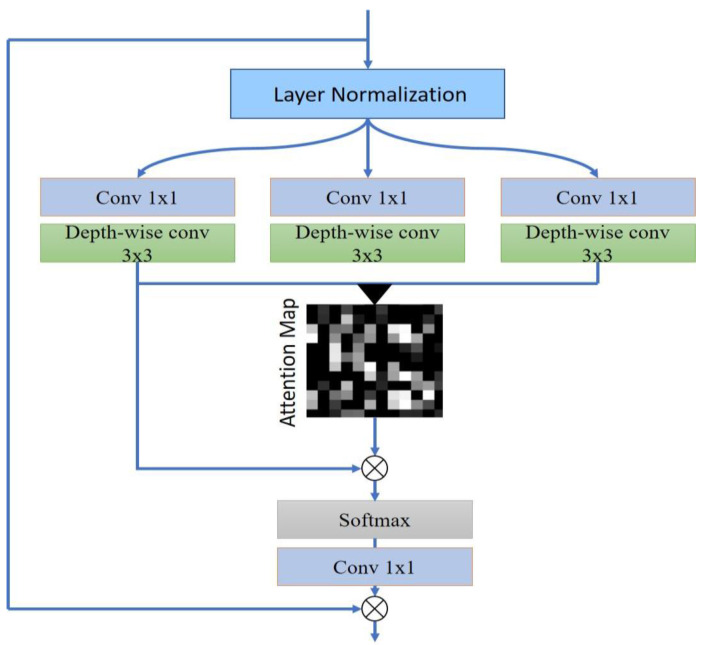
Illustration of the ADWC. This design addresses the computational challenges in standard attention networks, particularly for high-resolution medical images. Rather than focusing on spatial dimensions, ADWC emphasizes channel interactions, employing cross-covariance to generate an attention map that encapsulates the broader context. Through the integration of depth-wise convolutions, the model efficiently captures local nuances, culminating in a global attention map optimized for medical image denoising.

The reorientation of the attention mechanism to channel interactions and the linear complexity design of ADWC collectively contribute to a more efficient process. This efficiency does not come at the cost of performance. Instead, ADWC maintains and, in some aspects, enhances the quality of denoising in medical images, making it a suitable and innovative approach for handling the intricate requirements of medical image processing. To address this challenge, we introduce ADWC, depicted in Figure 3, designed with linear complexity. The fundamental approach shifts attention mechanisms from spatial dimensions to channels. Specifically, we calculate cross-covariance among channels to produce an attention map that implicitly captures the overall context. In ADWC, another crucial element, we incorporate depth-wise convolutions. This step accentuates the local context, forming a foundation for computing feature covariance that results in the global attention map, beneficial for medical image denoising. The ADWC process intricately balances the need for detail preservation and noise reduction. The method starts by calculating the cross-covariance among channels in the image data. Cross-covariance is a statistical measure that evaluates how much two different channels vary together, helping to establish the relationship between them. By computing these inter-channel relationships, ADWC effectively discerns which channels are most relevant to each other in the context of the image’s overall features. This step is crucial in medical imaging where understanding the interplay between different image aspects is essential for accurate diagnostics. Following the calculation of cross-covariance, ADWC incorporates depth-wise convolutions. Unlike traditional convolutions that mix inputs from all channels, depth-wise convolutions process each channel individually. This approach allows the method to concentrate on the local context within each channel, highlighting fine details and subtle variations in the image. In medical diagnostics, where even minor features can be significant, this focus on the local context is particularly beneficial.

The culmination of these processes leads to the formation of what is known as a global attention map. This map represents a weighted overview of the image, indicating which features across the channels warrant more focus. In the context of denoising, this global attention map is a strategic tool that guides the model to prioritize essential features while minimizing noise. It ensures that during denoising, vital details are not only preserved but also emphasized, thereby enhancing the overall quality and diagnostic utility of the medical image. The ADWC method stands out for its ability to intelligently and efficiently process medical images, balancing the preservation of crucial image details with effective noise reduction. The interplay of cross-covariance and depth-wise convolutions, leading to the creation of a global attention map, makes this approach particularly suited for the complex demands of medical image denoising.

Starting with a layer-normalized tensor *T* in $R^{H\times W\times C}$, our ADWC initially produces projections for inquiry (*I*), index (*X*), and data (*D*) that are enhanced with the local context. This enhancement is accomplished by using 1 × 1 convolutions to amass context across channels at the pixel level. Subsequently, 3 × 3 depth-wise convolutions are applied to capture the channel-specific spatial context in the student network. This results in the formulas:

(1) $$I=P_{w}^{I}D_{W}^{I}T$$

(2) $$X=P_{W}^{X}D_{W}^{X}T$$

(3) $$D=P_{W}^{D}D_{W}^{D}T$$

In the given context, $P_{W}$ represents the 1 × 1 point-wise convolution, while $D_{W}$ symbolizes the 3 × 3 depth-wise convolution. Within the network, we employ convolutional layers that are devoid of bias. Following that, we reconfigure the inquiry and index projections, so their dot–product interaction yields an attention map M with dimensions $R^{C\times C}$. This differs from the traditionally large attention map that has dimensions $R^{{\left(H\times W\right)}^{2}}$. In essence, the ADWC procedure can be described as:

(4) $$F^{\prime }=P_{wA}\left(\hat{I},\hat{X},\hat{D}\right)+F$$

In Equation (3) the resulting tensors *I*, *X*, and *D* represent different feature maps obtained after these convolutions. These feature maps are used to capture different aspects of the input data—*I* is the initial feature representation, *X* captures more complex interactions after further convolutions, and *D* captures depth-related features. Equation (4) describes how the feature map *F* is updated. *F*′ represents the new feature map after applying the attention mechanism, with *P_wA_* indicating the attention-based weight application to the feature maps *I*, *X*, and *D*. The original feature map *F* is adjusted by the weighted sum of the feature maps, leading to an enhanced feature representation that focuses on important features while ignoring irrelevant ones.

(5) $$A\left(\hat{I},\hat{X},\hat{D}\right)={\hat{D}}_{softmax}(\hat{X}*\hat{I}/l)$$

where *F* and *F*′ represent the input and output feature maps, respectively; $\hat{I}$ has dimensions $R^{H\times W\times C};$ $\hat{X}$ has dimensions $R^{\hat{H}\times \hat{W}\times \hat{H}}$; and $\hat{D}$ has dimensions $R^{\hat{H}\times \hat{W}\times \hat{H}}$. These matrices are derived by reshaping tensors from their original size. In this context, l acts as an adjustable scaling factor, determining the intensity of the dot product between $\hat{X}$ and $\hat{I}$ prior to the application of the softmax function. Mirroring the traditional multi-head attention approach, we segment the channel count into parts and concurrently train distinct attention maps for each. Together, these equations describe how different convolution operations and attention mechanisms are applied to the input data to produce an attention map that is sensitive to both local and global contexts, which is essential for effective medical image denoising. The process described captures complex dependencies among features, ensuring that the denoising process preserves important details while reducing noise.

### 2.4. Pruning Approach

After reparametrizing the student network, we introduce an iterative pruning approach. This method is designed to incrementally and systematically remove the least significant components of the network:

(6) $$N_{p}^{i}=t(p(N_{p}^{i-1},r))$$

The iterative pruning of the student network incorporates several components. *p* denotes the pruning operation, while *r* represents the pruning rate that dictates the fraction of components to be pruned in each iteration. The symbol *t* embodies the fine-tuning operation that ensues post-pruning, ensuring the model remains optimized. After each pruning operation, the network’s state is updated as $N_{p}^{i}$, marking its condition post the *i*-th pruning. Here, *p*, *r*, and *t* collectively ensure the optimization of the network post-pruning. The pruning operation, denoted by *p*, is the process where specific components or weights within the student network are systematically removed. This step is integral to reducing the network’s complexity, thereby enhancing its efficiency. The goal here is to streamline the network by eliminating elements that contribute less to the network’s performance, ensuring a leaner yet effective model. The pruning rate, represented by *r*, dictates the proportion of the network to be pruned in each iteration. This rate is a delicate balance; it must be carefully calibrated to ensure that while the network is being simplified, its core functional capabilities are not compromised. The pruning rate is central to the process, guiding the extent of simplification in each cycle and ensuring that the network remains robust and effective. Finally, the fine-tuning operation, symbolized by *t*, follows the pruning process. This step is crucial as it adjusts and optimizes the pruned network. Post-pruning, the network might lose some degree of accuracy or performance due to the removal of components. Fine-tuning addresses this by adjusting the remaining components, ensuring that the network continues to function optimally even after the pruning. It fine-tunes the network to adapt to its new, more streamlined structure. The iterative pruning process, through the harmonious functioning of *p*, *r*, and *t*, ensures that the student network remains efficient and optimized. While *p* and *r* work together to simplify the network, *t* ensures that this simplification does not come at the cost of the network performance, maintaining the delicate balance between efficiency and effectiveness.

Our method employs L2 filter pruning during the training process. This technique targets filters in the network based on their L2-norm values. Filters that possess smaller L2-norm values, suggesting lesser significance, are pruned first. As the iterative process progresses, the network undergoes a series of pruning and fine-tuning cycles. The pruning operation using *p* is applied at the rate *r*, followed by the *t* operation to fine-tune the model. This iterative approach continues until the network’s ability to predict effectively diminishes. When it is deduced that further pruning might adversely affect the model’s performance, the iterative process halts. The optimized state from the last effective pruning iteration is then selected as the final network configuration. Through this method, combining the principles of iterative pruning with the precision of L2 filter pruning, we aim to achieve an optimal balance, enhancing the network’s efficiency while preserving its predictive capability.

## 3. Experimental Results

In this segment, we showcase the empirical results achieved by our model when applied to actual denoising challenges.

### 3.1. Dataset

In our research, the efficacy of our model was assessed against three real-world denoising benchmarks: Chest X-rays dataset [27], Heart MRI dataset [28], AAPM dataset [29], and Abdomen CT dataset [30]. In the context of musculoskeletal radiographs, which inherently exhibit diverse image qualities and resolutions, the model developed in the paper employs several strategies to effectively handle these variations. Firstly, adaptive preprocessing plays a crucial role. The model uses sophisticated techniques to normalize differences in image quality and resolution. This involves enhancing image features that are crucial for accurate interpretation while mitigating issues like noise or poor contrast. By standardizing the images before they are fed into the model, we ensure that the algorithm’s performance is not hindered by variability in image quality. Secondly, the model is designed with robustness in mind. It leverages advanced machine-learning techniques that are less sensitive to variations in image quality. These algorithms can extract meaningful patterns and features from the radiographs, even when the image quality is suboptimal. This robustness is crucial for real-world application, where image quality can vary significantly. Furthermore, during the training phase, the model is exposed to a wide range of image qualities and resolutions. This exposure is critical for the model to learn how to handle the variability inherent in musculoskeletal radiographs. By training on a diverse dataset, the model becomes more adept at interpreting images with different qualities, ensuring consistent performance across a range of scenarios. Finally, ongoing evaluation and optimization are integral to the model’s approach. Regular assessments of the model’s performance with varying image qualities allow for continuous tuning and improvement. This iterative process ensures the model remains effective and accurate in clinical settings, where image quality can greatly impact diagnostic outcomes. The proposed model approach is multi-faceted, addressing the challenges posed by the diverse nature of musculoskeletal radiographs through a combination of advanced preprocessing, robust algorithmic design, comprehensive training, and continuous evaluation.

The Chest X-rays Dataset was drawn from the NIH collection by [27], boasting over 112,120 chest X-ray 2D images in PNG format. For the purposes of this study, the initial 5500 X-ray images were selected to train the MT-DDPM network, with each image resampled to a resolution of 256 × 256 pixels. During the training process, the intensity range of the dataset was normalized from [0, 255] to a spectrum of [−1, 1]. In the inference phase, the intensity range of synthetic images was confined to the [−1, 1] range.

Shifting focus to the Heart MRI dataset, we sourced 1902 2D heart MRIs from the Automated Cardiac Diagnosis Challenge (ACDC) dataset as outlined by [28]. These MRIs are in the form of axial slices that span from the heart’s base to the left ventricle, exhibiting spacings between 5 to 8 mm. After being extracted, these 2D slices underwent a resampling process to 2 × 2 mm, subsequently being adjusted to secure a consistent resolution of 256 × 256 pixels. The training process involved normalizing scan intensities to fit within the [−1, 1] range.

CNN denoising architectures were educated utilizing images sourced from the Mayo/AAPM Low-Dose CT Grand Challenge collection. This compilation encompasses full-dose (FD) abdominal CT visuals from 30 subjects, paired with their analogous simulated quarter-dose (QD) CT imagery. Every piece of training data underwent reconstruction, featuring a 340 mm field of view, a mid-range smooth kernel (B30), coupled with a slice thickness of 1.0 mm and a 0.8 mm interval.

Lastly, the Abdomen CT Dataset featured 2178 abdomen CT slices from the Beyond the Cranial Vault (BTCV) dataset, as introduced by [30]. This collection consisted of 30 patient scans. After an initial resampling process, each axial slice attained a resolution of 256 × 256 pixels. The research team curated a subset from this dataset, including only slices displaying at least one organ. The normalization and attenuation coefficient value strategies used in the AAPM dataset were equivalently applied here during training and inference.

The use of a diverse range of datasets for training and testing is a fundamental approach to promote generalizability. By incorporating various types of medical images such as chest X-rays, heart MRI, AAPM, and abdomen CT scans, the model is exposed to a wide spectrum of data characteristics. This diversity in training helps the model learn to identify and adapt to the unique features and types of noise present in different imaging modalities. The architecture of the model itself, featuring components like the NoiseContextNet Block and the ADWC, is designed to be adaptable and robust. These components are structured to focus on identifying and processing features that are critical for noise reduction, irrespective of the specific type of medical image. Such an architecture is more likely to perform consistently across different datasets, as it focuses on general principles of noise and image characteristics rather than being tailored to specific types of images. Moreover, the methodological approach, which includes extensive testing and comparative analysis, also plays a crucial role. By evaluating the model’s performance using standard metrics like PSNR and SSIM across various datasets, the researchers can assess and ensure its effectiveness and reliability in diverse scenarios. Finally, the iterative pruning technique used post-reparameterization in the student network might contribute to the model’s ability to generalize. By streamlining the model and reducing its complexity, it is more likely to focus on the most salient features that are relevant across different types of medical images, thereby enhancing its general applicability. Together, these approaches form a comprehensive strategy to ensure that the model can effectively denoise a wide range of medical images, making it a versatile tool in the field of medical imaging.

### 3.2. Implementation Details and Metrics

Our training experiments were conducted utilizing NVIDIA 3080ti hardware. During the training phase, image augmentations involving random flips and rotations were employed to enhance the dataset’s diversity. For optimization purposes, we adopted the Adam optimizer. When progressing through the training of the teacher network and subsequently distilling knowledge to the student network, we initiated with a learning rate of 1 × 10^−4^. This rate was reduced by half after every 100,000 iterations. Throughout this phase, the L1 loss was the chosen metric for supervision. In refining the distilled network using a progressive learning approach, we started with a learning rate of 2 × 10^−5^, which was then halved at intervals of every 20,000 iterations.

The evaluation of our methodologies was anchored in widely accepted metrics, specifically the peak signal-to-noise ratio (*PSNR*):

(7) $$PSNR=10*{log}_{10}\left(\frac{{\left(2^{n}-1\right)}^{2}}{MSE}\right)$$

and the Structural Similarity Index Measure (*SSIM*):

(8) $$SSIM(x,y)=\frac{\left(2\mu_{x}\mu_{y}+C_{1})(2\sigma_{x}\sigma_{y}+C_{2}\right)}{(\mu_{2}^{x}+\mu_{2}^{y}+C_{1})\left(\sigma_{2}^{x}+\sigma_{2}^{y}+C_{2}\right)}$$

Both metrics served as indicators of the efficacy and accuracy of the denoising processes implemented.

### 3.3. State-of-the-Art Models

Table 1 provides a detailed comparative analysis of average PSNR and SSIM scores, delineating the performance of diverse image-denoising models across a spectrum of medical imaging datasets. The ADL model [15] exhibits commendable stability in its PSNR performance, particularly distinguishing itself in the Heart MRI dataset with a PSNR of 34.02. In stark contrast, the CNNCT model [13] lags notably behind, with its PSNR scores dipping as low as 20.14 in the AAPM dataset. The ODVV model [31] stands out with its higher PSNR scores, especially a notable 35.32 in the Abdomen CT dataset. Meanwhile, StruNet [32] offers competitive results, particularly in SSIM, where it achieves a remarkable 94.15% in the Abdomen CT dataset. However, it is the proposed model that truly excels, registering the highest PSNR score of 35.89 for abdomen CT and leading with the highest SSIM scores across all datasets, notably reaching 96.57% for abdomen CT. These figures not only underscore the proposed model’s proficiency in noise reduction but also its adeptness at preserving structural integrity across various imaging modalities. The proposed model’s performance suggests a significant advancement in the denoising domain, positioning it as a versatile and powerful tool for medical image processing.

### 3.4. Visual Comparison

**Figure 4:**
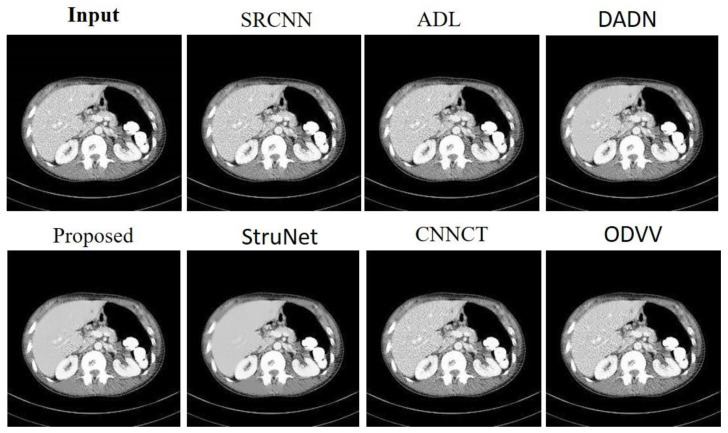
Results achieved by the evaluated noise-reduction models on the AAPM dataset.

Figure 4 provides a visual comparison of denoising methods on an abdominal CT image from the AAPM dataset. The input image serves as the baseline, displaying the raw scan with its inherent noise and details. The SRCNN [33] method shows a little noise reduction, but the image remains relatively grainy. The ADL [15] approach appears to reduce noise while maintaining a good level of detail, though some structures are not as crisp. DADN [22] further smoothens the image, with noticeable improvement in noise suppression, yet at the risk of losing fine details. The lower row begins with the proposed method, which visibly enhances the image by reducing noise substantially without compromising the integrity of the anatomical structures. The clarity of internal features is markedly improved, making this method potentially valuable for clinical evaluation. StruNet [31] also offers a cleaner image, but with a slight blur that could obscure finer details. CNNCT [13] rendition is cleaner than the input but does not reach the level of noise reduction achieved by the proposed method. Lastly, ODVV [32] provides a denoised image, yet some areas appear over-smoothed, which could be problematic for diagnostic purposes. The proposed model stands out with its balance between noise reduction and detail preservation, suggesting its superior capability for producing diagnostically useful images.

**Figure 5:**
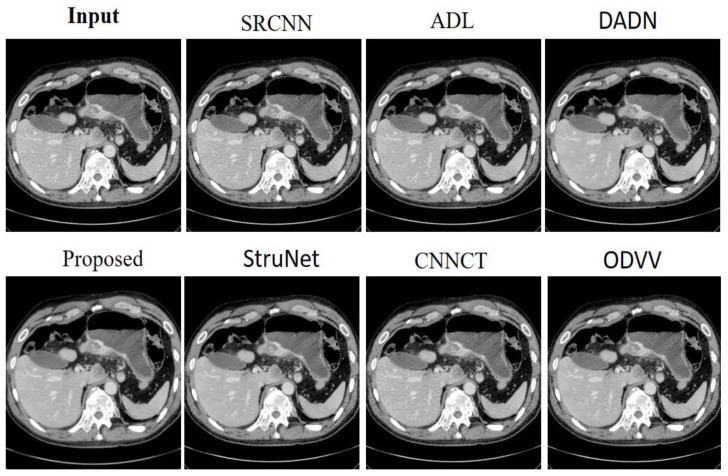
Outcomes derived from assessing noise-reduction algorithms on the Abdomen CT dataset.

Figure 5 presents a visual assessment of several denoising techniques as applied to an abdominal CT scan from the AAPM dataset. The SRCNN [33] model shows some improvement in clarity over the input, yet some noise is still perceptible. ADL [15] method improves upon this, smoothing out more noise while preserving essential details. DADN [22] attempt at denoising is more aggressive, potentially at the expense of finer structures which seem slightly diminished. The proposed method, showcased in the second row, impressively refines the image, achieving a significant reduction in noise while maintaining the sharpness of anatomical features. StruNet follows, offering a denoised image, but with a softness that suggests some loss of fine detail. The CNNCT [13] model provides a cleaner version than the original scan but does not quite match the noise suppression displayed by the Proposed technique. ODVV [32] output shows a well-denoised image, yet certain textures appear somewhat flattened, which could impact the diagnostic value. The proposed method excels, striking an optimal balance between noise removal and detail retention, suggesting its effectiveness for enhancing diagnostic imaging in a clinical setting.

**Figure 6:**
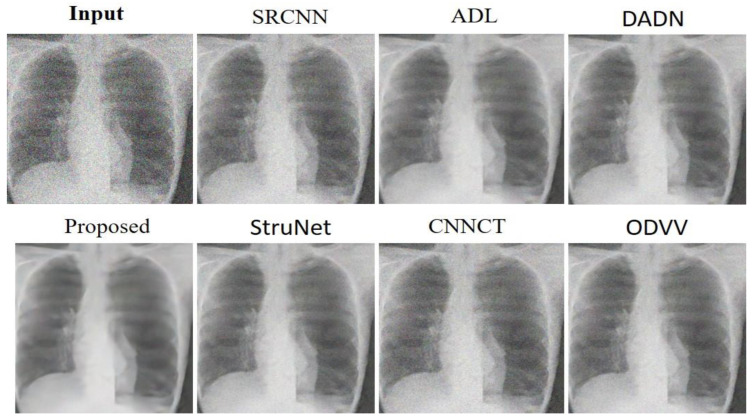
Results were gathered from the noise-removal models applied to the Chest X-ray dataset.

In Figure 6 the SRCNN [33] method provides a visible improvement in image quality with reduced noise, although the finer details are not entirely clear. ADL [15] technique further enhances image clarity, but there are some finer structures that are not as sharply defined as they could be. DADN [22] appears to strike a balance between noise reduction and detail preservation, offering a clearer image while maintaining important anatomical features. The proposed method demonstrates a significant advancement in denoising, presenting a much clearer image where anatomical structures are more distinguishable, and noise is substantially reduced. This suggests an effective preservation of important clinical details. StruNet approach also reduces noise, yet the image appears slightly less sharp when compared to the proposed method. CNNCT [13], while offering a denoised image, does not achieve the same level of clarity and noise suppression as the proposed method. ODVV [32] delivers a denoised image but with a potential compromise in the visibility of finer details. The proposed method outperforms the other techniques in terms of denoising effectiveness, presenting a clean image that retains critical anatomical details.

**Figure 7:**
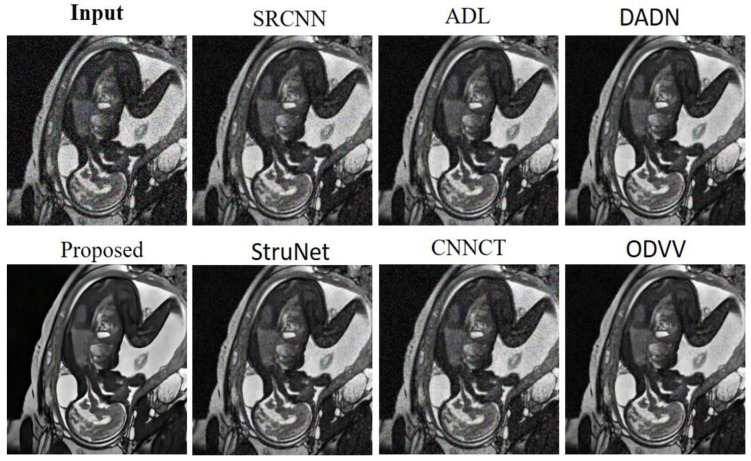
Outcomes from the denoising models tested on the Heart MRI dataset.

In Figure 7, the SRCNN [33] model offers some denoising benefits, clearing up a portion of the noise but not to the extent required for fine diagnostic detail. The ADL [15] model improves upon this, reducing noise further and beginning to clarify some of the more intricate structures within the image. The DADN [22] output suggests a more aggressive denoising approach, which while reducing noise, also suppresses some of the subtle features that are often crucial for accurate medical interpretation. The proposed model appears to significantly enhance the image quality, striking an excellent balance between noise reduction and the preservation of essential details within the image. This level of clarity is beneficial in identifying specific medical conditions from the scan. StruNet performs adequately in noise suppression but slightly blurs finer details when compared to the proposed model. CNNCT [13] rendition shows improved clarity over the input, yet it does not match the proposed model’s balance of denoising and detail retention. ODVV [32] provides a cleaner image, but there is a noticeable loss of detail that is detrimental for fine-grained analysis. The proposed model rendering stands out, potentially offering clinicians a more defined and diagnostically useful image, free from the distractions of noise yet rich in detail crucial for patient assessment and subsequent medical decision-making.

## 4. Conclusions

In light of the data presented in Table 1 and the underlying methodology described, the significance of medical image denoising for precise diagnostics is reiterated. The persistent dilemma of harmonizing image quality with computational thriftiness is evident. However, this study’s avant-garde approach, which emphasizes refined training paradigms without heavily leaning on intricate network modifications, offers a promising direction. The integration of the teacher–student network design with staggered distillation provides an advantageous balance between operational swiftness and the capability to discern intricate details. This is aptly complemented by the NoiseContextNet Block, which adeptly differentiates genuine anatomical details from noise-afflicted zones. The deployment of the attention-based ADWC introduces a refined attention shift, creating a holistic attention blueprint indispensable for proficient denoising. The culmination of these features, in conjunction with the iterative pruning technique, post-reparameterization, sets forth a methodology that not only maintains the model’s predictive acumen but also augments its computational efficiency. This confluence of techniques and the resulting performance, as substantiated by Table 1, underscores the viability and superiority of the proposed model in the domain of medical image denoising. However, we acknowledge certain limitations in our work. The model complexity, while manageable, requires a degree of computational resource that may not be universally accessible. Moreover, the depth of denoising achieved, while beneficial for current imaging modalities, needs to be tested against an ever-evolving array of medical imaging technologies to ensure continued relevance. The implications of our work for the field are multifaceted. Not only does it offer a practical tool for current practitioners, enhancing the quality of diagnostic imaging, but it also provides a methodological framework upon which future research can build. Our approach can serve as a benchmark for the development of even more advanced denoising techniques, potentially incorporating emergent technologies such as deep learning and artificial intelligence.

In terms of future work, we aim to explore the integration of our model with real-time imaging systems, assess its adaptability to different imaging modalities beyond those tested, and fine-tune its computational efficiency. We also see substantial scope for incorporating feedback mechanisms that allow the model to learn from diagnostic outcomes, thereby continually refining its denoising capabilities. Through continued research and development, we strive to ensure that our contributions remain at the forefront of the field, driving advancements in medical imaging technology.

## 5. Discussion

Our study marks a significant leap in medical image denoising by introducing a pioneering teacher–student network model. This model stands as a testament to innovation in the field, enhancing the denoising process not just in the quality of output but also in operational efficiency. The novelty of our approach lies in its unique application of the teacher–student paradigm, a first in the realm of medical imaging. This architecture redefines knowledge transfer and model optimization in image processing. At the heart of our method is the NoiseContextNet Block, a bespoke component meticulously crafted to detect and mitigate a variety of noise types that are commonly encountered in medical images. The ingenuity of the NoiseContextNet Block lies in its advanced algorithms, which are specifically tailored to understand and address the nuanced challenges posed by different noise patterns. This focus on contextual noise identification and reduction is a novel stride in ensuring that the clarity of medical images is substantially improved. Moreover, our approach integrates an iterative pruning technique, a strategic innovation aimed at refining the efficiency of the model. This technique is not merely a functional improvement but a critical step towards rendering our solution practical for real-world medical imaging scenarios. It ensures that our model is not only effective but also viable within the computational constraints of medical imaging environments.

The teacher–student network design inherently contributes to reducing overfitting. In this design, the student network learns from a simplified representation of the teacher network’s knowledge. This approach encourages the student model to focus on generalizable features rather than memorizing the specific details of the training data. The introduction of the Iterative Pruning Technique after reparameterizing the student network is another critical factor. This technique involves systematically removing less significant connections within the network. By doing so, it not only streamlines the proposed model, making it more efficient, but also helps in mitigating overfitting. Pruning reduces the model’s complexity, preventing it from fitting too closely to the noise or idiosyncrasies in the training data. ADWC also plays a role in addressing overfitting. By shifting focus from spatial dimensions to channels, it allows the model to prioritize learning features that are more relevant across different datasets. This aspect of the network design can help in enhancing the model’s ability to generalize better. The combination of these strategies in the teacher–student network model, particularly in the context of a large feature set, is essential in ensuring that the model learns to extract and utilize the most relevant features for denoising, without overfitting to the specifics of the training dataset.

The contributions of our proposed solution extend beyond mere technical improvements. They represent a holistic advancement in medical image processing, combining state-of-the-art techniques with practical applicability to set new benchmarks in image clarity and diagnostic accuracy.

## Figures and Tables

**Table 1:** Average PSNR (in dB) and SSIM scores with their standard deviations for various techniques on image datasets are presented. The top-performing method is emphasized in bold.

Models	PSNR				SSIM			
Datasets	Chest X-rays	Heart MRI	AAPM	Abdomen CT	Chest X-rays	Heart MRI	AAPM	Abdomen CT
ADL [15]	34.05	34.02	23.7	32.54	91.99%	89.85%	78.36%	90.25%
CNNCT [13]	28.24	26.57	20.14	27.45	89.21%	85.44%	69.89%	86.59%
DADN [22]	34.13	33.28	24.88	34.75	91.79%	90.62%	76.88%	93.85%
ODVV [32]	35.13	33.75	24.17	35.32	91.72%	89.54%	79.10%	92.28%
StruNet [31]	32.12	34.74	24.99	34.99	91.45%	90.71%	**78.88%**	94.15%
SRCNN [33]	26.95	27.12	20.74	25.62	79.99%	86.77%	68.47%	85.76%
Proposed	**35.32**	**34.99**	**25.85**	**35.89**	**95.85%**	**93.49%**	**79.23%**	**96.57%**

## Data Availability

All data Open Access.
